# *Glutathione* *S-transferase* Polymorphisms in Head and Neck Squamous Cell Carcinoma Treated with Chemotherapy and/or Radiotherapy

**DOI:** 10.31557/APJCP.2020.21.6.1637

**Published:** 2020-06

**Authors:** Mauricio Pereira Maniglia, Anelise Russo, Patrícia Matos Biselli-Chicote, Juliana Garcia de Oliveira-Cucolo, Gabriela Helena Rodrigues-Fleming, José Victor Maniglia, Érika Cristina Pavarino, Eny Maria Goloni-Bertollo

**Affiliations:** 1 *Department of Molecular Biological, Genetics and Molecular Biology Research Unit – UPGEM, Faculty of Medicine of São José do Rio Preto – FAMERP, São José do Rio Preto, São Paulo, Brazil. *; 2 *Department of Otolaryngology and Head and Neck Surgery, Sao Jose do Rio Preto Medical School, São José do Rio Preto, São Paulo, Brazil. *

**Keywords:** Chemotherapy agents, genetic polymorphism, glutathione transferase, head and neck neoplasms

## Abstract

**Background/Aim:**

The Glutathione S-transferases (GSTs) are important carcinogen-metabolizing enzymes. Polymorphisms involved in these enzymes can modulate the development and treatment of head and neck cancer. To investigate the association of *GSTs* polymorphisms with head and neck cancer and risk factors, clinical-pathological features, and survival time of the patients treated with chemotherapy and/or radiotherapy.

**Methods:**

The *GST* gene polymorphisms were evaluated in 197 cases and 514 controls by PCR-RFLP-Polymerase Chain Reaction Restriction Fragment Length Polymorphism.

**Results:**

The *GSTP-313* was associated with a decreased risk for HNSCC (p=0.050). The *GSTP1 *haplotype analysis revealed a higher frequency of the AC and AT haplotypes in the case group than in the control group (p=0.013 and p=0.019, respectively), and the opposite for G-C haplotype (p = 0.015). Yet, the different combinations between the genotypes were associated with an increased risk of cancer. The study showed no association between the polymorphisms and primary tumor site, clinical-pathological characteristics, treatment (chemotherapy and/or radiotherapy) and survival time of the patients.

**Conclusion:**

The* GST* polymorphisms combination showed an increased risk for carcinogenesis, and studies with larger casuistry can contribute to the clarification of the role in individual patient differences for the response to chemotherapy and/or radiotherapy and identify biomarkers of susceptibility.

## Introduction

Head and neck cancer presents an estimated 834,860 new cases in the world for 2018/2019 and comprises a heterogeneous group of tumors that originate in the squamous cells of the upper digestive tract lining epithelium, including lip, oral cavity, nasal cavity, paranasal sinus, pharynx, larynx (IARC, 2019). It is a multifactorial disease, influenced by age, gender, environmental factors such as smoking habits, alcohol consumption, HPV infection, genetic factors such as gene polymorphisms and exposure to radiation solar UVA (lips cancer) (Mirghani et al., 2017). 

Regarding genetic factors, studies show that polymorphisms in genes that encode drug metabolism enzymes used in chemotherapy can cause variability in drug responses and modulate treatment outcomes in head and neck squamous cell carcinoma (HNSCC) cases, thus individualized treatment can improve the better patient response (Ruwali et al., 2009; Galbiatti et al., 2013; Carron et al., 2017; Hasegawa et al., 2018; Macedo et al., 2019). 

Drug metabolism or xenobiotic metabolism involves two major types of enzymes. Conjugated enzymes/Phase II involves conjugation with an endogenous substrate (glutathione, sulfate, glucose, acetate) by glutathione-S-transferase (GSTs) enzymes. GSTs act as inactivating enzymes for Phase I products/mediated oxidative metabolism by cytochrome P450 and microsomal epoxide hydrolase, leading to hydrophilic metabolite capable of being excreted for homeostatic control and detoxification of lipophilic xenobiotics which occurs in the Phase III (Marchewka et al., 2017).

Genetic variations in glutathione S-transferases have been associated with changes in the catalytic activities of the enzyme and can be associated with increased DNA adducts and increasing the risk of cancer (Chatterjee and Gupta, 2018; Singh and Ghosh, 2019; Zhang et al., 2019). Also, low response to chemotherapy applying cisplatin for oral cancer was found in cases of *GSTP1* AA313 wild genotype, demonstrating the possible role of these polymorphisms in patient responses to chemotherapy (Ruwali et al., 2009).

Thus, studies concerning these polymorphisms are important to identify biomarkers of susceptibility to HNSCC. Considering the evidence presented, this study aimed to investigate the association of *GSTM1* and *GSTT1* null genotypes and single nucleotide polymorphisms *(SNPs) A313G* and *C341T *of the *GSTP1 *gene with head and neck cancer, as well as to verify the association between these polymorphisms and the anatomical site of tumor occurrence, clinical-pathological characteristics, relapse-free time and survival of patients with head and neck cancer treated with chemotherapy and/or radiotherapy.

## Materials and Methods

*Patients and Methods*

*Sample characterization*

A total of 711 individuals were included in the present retrospective case-control study, 197 patients composing the case group, diagnosed with HNSCC during follow-up at the Otorhinolaryngology and Head and Neck Surgery Service belonging to the FAMERP/FUNFARME hospital medical complex, in São José do Rio Preto – SP, and 514 healthy individuals with no history of neoplasia obtained at the São José do Rio Preto Base Hospital blood center, comprising the control group. Patient and control data were collected through standardized questionnaires. All samples were obtained after the signing of an Informed Consent Term. The study was approved by the Research Ethics Committee (No. 697,896).

The inclusion criterion for the case group was pathological biopsy confirmation of primary tumors diagnosed as HNSCC treated with chemotherapy and/or radiotherapy at the reference hospital in Southwest São Paulo by FAMERP/FUNFARME Otorhinolaryngology, Head and Neck Surgery of Services. Exclusion criteria for the case group were patients with head and neck carcinomas other than squamous cell carcinoma and patients who were not submitted to chemotherapy and/or radiotherapy.

Regarding the control group, the inclusion criteria were healthy individuals aged ≥40 years regardless of gender and without cancer or any family history of neoplasia. Exclusion criteria for the control group were individuals with health-related complaints, changes in blood counts, infectious-contagious diseases, patients presenting chronic diseases, a family history of cancer and <40 years of age.

Subjects who had smoked >100 cigarettes during their lifetime were considered smokers, and subjects who consumed more than four alcohol doses per week (one dose corresponding to liquor – 30 mL, glass of wine – 102 mL containing 12% of alcohol, or can of beer – 340 mL) were considered alcohol consumers. The analyzed variables were gender, age, smoking habit, alcohol consumption, primary site and clinical-pathological features of the tumor. Patient treatment included radiotherapy and/or chemotherapy and the main chemotherapeutic agents were cisplatin, methotrexate, and 5-fluorouracil.

*Clinical characterization*

The neoplasias were categorized concerning the primary site, namely oral cavity, pharynx, and larynx. American Joint Committee for Cancer – AJCC (Edge and Compton, 2010) and Union for International Cancer Control – UICC (Sobin, 2011) criteria for tumor classification involve tumor extension (T), nodal metastasis (N) and distant metastasis (M). T classification was categorized as smaller (T1, T2) and larger (T3, T4) tumors. N classification was defined as the absence (N0) and presence (N1, N2, N3) of nodal metastasis. M classification was dichotomized into absence (M0) or presence (M1) of distant metastasis. The stages were divided into early (stages I and II) and advanced disease (III and IV) (Lee, 2011; Sobin, 2011). Diagnosis, primary anatomical site, TNM classification, and certain clinical information were obtained from patient medical records, included in the study and evaluated by the study author (an otorhinolaryngologist and head and neck surgeon).

*Molecular analysis*

Genomic DNA was extracted from total peripheral blood, according to Salazar (Salazar et al., 1998). *GSTT1 *and *GSTM1* polymorphisms: performed by Multiplex Polymerase Chain Reaction (PCR), using the *CYP1A1* gene as an internal positive control for primer amplification (Abdel-Rahman et al., 1996). PCR products were analyzed on 1.5% agarose gels stained with ethidium bromide.

A313G polymorphism of the *GSTP1 *gene: The PCR products, 176 base pair (bp) were treated with the *Bsmal* enzyme for application of the PCR-RFLP technique: AA wild genotype (176bp); GG polymorphic genotype (91 and 85 bp) and AG heterozygous (176, 91 and 85 bp) (Harries et al., 1997). C341T polymorphism of *GSTP1* gene: The PCR products, 420 base pair (bp) were treated with *Acil* for application the PCR-RFLP technique: CC wild genotype (247, 118 and 55 bp); CT heterozygous genotype (365, 247, 118 and 55 bp) and TT polymorphic genotype (365 and 55 bp) (Watson et al., 1998). The enzymatic digestion products were then analyzed after electrophoresis on 2.5% agarose gels stained with ethidium bromide. The molecular evaluation was repeated in 10% of the samples to confirm the genotyping of the investigated polymorphisms.

*Statistical analysis*

Genotypic frequencies of the A313G (rs1695) and C341T (rs749174) *GSTP1* polymorphisms were evaluated for the Hardy-Weinberg Equilibrium (HWE) by the Chi-Square Test (*χ*^2^) using the BioEstat program, version 5.0. The haplotype analysis of the A313G and C341T GSTP1polymorphisms was performed using the Haploview program, version 4.2. The SNPStats program (available at http://bioinfo.iconcologia.net/SNPstats_web) was used to perform the multiple logistic regression to evaluate the effect of the variables analyzed and cancer association between the genetic models of the polymorphisms and the development of HNSCC, adjusted for age, gender, and smoking and drinking habits. The analysis included age (reference: <median of the case group), gender (reference: female), smoking habit (reference: non-smokers) and alcohol consumption (reference: non-consumers). The effect of the polymorphisms was evaluated in the models as (1) codominant (heterozygous vs wild-type homozygous and polymorphic homozygous vs wild-type homozygous); (2) dominant (heterozygous + polymorphic homozygous vs wild-type homozygous); (3) recessive (homozygous polymorphic vs wild- type homozygous + heterozygous); (4) overdominant (heterozygous vs wild-type homozygous + polymorphic homozygous); or (5) additive (polymorphic homozygous with 2 + heterozygous vs wild-type homozygous). The Instat Program was used to evaluate the association of the combined GSTT1, GSTM1, GSTP1 A313G* and GSTP1 C341T* polymorphisms in HNSCC risk.

Concerning size (T), tumors were classified as T1 and T2 (small) and T3 and T4 (large). According to regional lymph node involvement (N), cases were classified as negative (N0) or positive (N1, N2, and N3). Tumors were classified as M0 (absence of distant metastases) and M1 (with metastases) regarding metastasis. Polymorphism effects on relapse and survival time of patients presenting HNSCC were analyzed using the Kaplan Meier Curve and Log Rank test. The results were presented as the Odds Ratio (OR) and 95% confidence interval (CI - 95%). The level of significance was established as 5% (p ≤ 0.05).

## Results

A total of 197 patients and 514 controls were analyzed. The median age was 59 years old, ranging from 34 to 83 in the case group and 44 years old, ranging from 24 to 90 in the control group. Males were predominant in both case (90.0%) and control (71.8%) groups. The Multiple Logistic Regression analysis for socio-demographic characteristics and risk factors showed that in the Case group male gender (OR = 2.13; 95% CI = 1.03 - 4.38; p = 0.040), age ≥ 59 years (OR = 11.66; 95% CI = 6.51 - 20.86; p < 0.001), smoking habit (OR = 11.00; 95% CI = 5.71 - 21.20; p < 0.001) and alcoholic habit (OR = 4.14; 95% CI = 2.25 – 7.64; p < 0.001) were associated with greater susceptibility to the development of this neoplasm (Table 1).

In the Case group, the genotype frequencies of *GSTP1*-A313G were 56.7% for the A/A, 34.8% for A/G and 8.4% for G/G, while in the control group, these frequencies were 44.2%, 43.0%, and 12.8%, respectively. As showed in the Table 1, the *GSTP1*-313 A/G + G/G genotypes was associated in the dominant model with a decreased risk for HNSCC (OR = 0.64; 95% CI = 0.42 – 1.00; p = 0.050). In contrast, for *GSTM1* and *GSTT1* null genotype and SNP *GSTP1*-C341T, no significant differences were found in the evaluated two groups. 

We evaluated the combined effect of the four polymorphisms studied (Table 2), an increase risk of HNSCC was observed in the presence of the duple combination: *GSTT1* non-null/*GSTM1* null genotypes (OR = 1.93; 95%CI: 1.15-3.24; p = 0.012), GSTP1-313AA/-341CT+TT (OR = 6.12; 95%CI: 1.87-20.04, p = 0.001); triple combination: *GSTT1* non-null/GSTM1 null/GSTP1-313AA (OR = 3.33; 95%CI: 1.57-7.05, p = 0.002), GSTT1 null/GSTM1 non- null/GSTP1-313AA (OR = 2.10; 95%CI: 1.21-3.63, p = 0.008), *GSTT1* non-null/*GSTM1*non- null/*GSTP1*-313AA (OR = 2.43; 95%CI: 1.10-5.37, p =0.042), *GSTT1* non-null/*GSTM1* null/*GSTP1*-341CC (OR = 1.98; 95%CI: 1.14-3.44, p = 0.017), and fourth combination: *GSTT1 *null/GSTM1 null/GSTP1-313AA/GSTP1-341CT+TT (OR = 7.52; 95%CI: 1.30-43.45, p = 0.025), *GSTT1* null/*GSTM1* non-null/*GSTP1*-313AA/*GSTP1*-341CT+TT (OR = 15.04; 95%CI: 1.60-140.68, p = 0.010) and *GSTT1 *non-null/*GSTM1* null/*GSTP1*-313AA/*GSTP1*-341CC (OR = 2.86; 95%CI: 1.30-6.28, p = 0.010). 

Genotypic frequencies for the A313G and C341T *GSTP1* polymorphisms were in Hardy-Weinberg equilibrium in both groups (p > 0.05). The A313G/C341T *GSTP1* haplotypes (Table 3) revealed a higher frequency of the two wild alleles (A-C) in the case group than in the control group (72.0 and 65.0, respectively; p = 0.013). Similarly, a higher frequency of the A-T haplotype was also found in the case group than in the control group (3.0 and 0.9, respectively; p = 0.019). However, the opposite was observed for the G-C haplotype, which was more frequent in the control group than in the case group (23.0 and 30.0, respectively; p = 0.015).

Regarding the anatomical site, 44.0% of the tumors were found in the oral cavity, 32.0% in the larynx, 19.0% in the pharynx, and 5.0% of the individuals presented tumors of unknown origin. Considering tumor size, 57.0% of the patients presented large tumors (six tumors were not possible to classify in T categories). No involvement of regional lymph nodes (N0) was observed in 55% of the cases (two tumors were not possible to classify in N categories). Only 3.0% of patients presented distant metastases (M1). The staging was evaluated for 194 patients. The present study demonstrated no association between the investigated polymorphisms and primary tumor site (Table 4), as well as between the genotypes and the clinical-pathological tumor characteristics (Table 5). Analyzing patient treatment, the most common was a radiotherapy and chemotherapy association with 69.0%, against 26.0 and 5.0% for exclusive radiotherapy and exclusive chemotherapy, respectively.

The Kaplan-Meier Curve analysis indicated that relapse-free and survival time in patients with *GSTM1*, or *GSTT1*, or *GSTP1* polymorphism presented no significant difference concerning allele carriers and non-carriers patients (Table 6).

**Table 1:** Sociodemographic Characteristics, Risk Factors, and Polymorphisms, *GSTP1 A313G, GSTP1 C341T, GSTT1* and *GSTM1* in Patients with Head and Neck Cancer and Controls

Variables	Case n=197 (%)	Control n=514 (%)	OR (95% CI)	*P-value*
Gender				
Female	19 (10.0)	145 (28.0)	1	0.040
Male	178 (90.0)	369 (72.0)	2.13 (1.03-4.38)	
Age (Median)				
<59	97 (49.0)	448 (87.0)	1	
≥59	100 (51.0)	66 (13.0)	11.66 (6.51-20.86)	<0.001
Smoking Habit				
Non-smoke	20 (10.0)	309 (60.0)	1	<0.001
Smoke	177 (90.0)	205 (40.0)	11.00 (5.71-21.20)	
Alcoholic Habit				
Non-alcoholic	28 (14.0)	260 (51.0)	1	<0.001
Alcoholic	169 (86.0)	254 (49.0)	4.14 (2.25-7.64)	
Polymorphisms				
*GSTT1*				
+/+	140 (71.1)	402 (78.2)	1	0.610
0/0	57 (28.9)	112 (21.8)	1.14 (0.69-1.87)	
*GSTM1*				
+/+	98 (49.8)	275 (53.5)	1	0.400
0/0	99 (50.2)	239 (6.5)	1.20 (0.78-1.85)	
*GSTP1-A313G* ^a^				
Codominant				
A/A	101 (56.7)	227 (44.2)	1	0.140
A/G	62 (34.8)	221 (43)	0.66 (0.41-1.05)	
G/G	15 (8.4)	66 (12.8)	0.61 (0.29-1.25)	
Dominant				
A/A	101 (56.7)	227 (44.2)	1	0.050
A/G-G/G	77 (43.3)	287 (55.8)	0.64 (0.42-1.00)	
Recessive				
A/A-A/G	163 (91.6)	448 (87.2)	1	0.360
G/G	15 (8.4)	66 (12.8)	0.73 (0.36-1.46)	
Overdominant				
A/A-G/G	116 (65.2)	293 (57.0)	1	0.16
A/G	62 (34.8)	221 (43.0)	0.72 (0.46-1.14)	
Additive	---	---	0.74 (0.53-1.02)	0.062
*GSTP1-C341T* ^b^				
Codominant				
C/C	173 (89.6)	460 (89.5)	1	0.260
C/T	20 (10.4)	50 (9.7)	0.80 (0.40-1-60)	
T/T	0 (0)	4 (0.8)	0	
Dominant				
C/C	173 (89.6)	460 (89.5)	1	0.380
C/T - T/T	20 (10.4)	54 (10.5)	0.74 (0.37-1.46)	
Recessive				
C/C - C/T	193 (100)	510 (99.2)	1	0.150
T/T	0 (0)	4 (0.8)	0	
Overdominant				
C/C - T/T	173 (89.6)	464 (90.3)	1	0.550
C/T	20 (10.4)	50 (9.7)	0.81, (0.41-1.62)	
Additive	---	---	0.70 (0.37-1.33)	0.270

^a^, Odds Ratio (OR) adjusted for age, gender, and alcohol and smoking habits and polymorphisms significant p values for p <0.05 ^a ^Amplification was possible for 692 individuals of the case group ^b^Amplification was possible for 707 individuals of the case groupd.

**Table 2:** Association of the Combined *GSTT1, GSTM1, GSTP1* A313G* and GSTP1 C341T* Genotypes in Patients with Head and Neck Cancer and Controls, Adjusted for Gender, Age, Smoking and Alcohol Consumption

*GSTT1*	*GSTM1*			Case (187)	Control (514)	OR (95%IC)	P value
Positve	Positive			66	220	1	
Positive	Negative			74	182	1.355 (0.92-1.99)	0.140
Negative	Positive			32	55	1.939 (1.158-3.247)	0.012
Negative	Negative			25	57	1.462 (0.847-2.521)	0.191
*GSTP1* A313G*	*GSTP1* C341T*			Case (178)	Control (514)	OR (95%IC)	P value
AA	CC			91	223	1	
AA	CT/TT			10	4	6.12 (1.87-20.04)	0.001
AG/GG	CC			67	237	0.69 (0.48-0.99)	0.057
AG/GG	CT/TT			10	50	0.49 (0.23-1.00)	0.056
*GSTT1*	*GSTM1*	*GSTP1 *A313G*		Case (178)	Control (514)	OR (95%IC)	P value
Positve	Positive	AA		29	96	1	
Positive	Positive	AG/GG		28	124	0.97 (0.54-1.73)	1
Negative	Positive	AA		17	22	3.33 (1.57-7.05)	0.002
Negative	Positive	AG/GG		13	33	1.69 (0.79-3.62)	0.214
Positive	Negative	AA		42	86	2.10 (1.21-3.63)	0.008
Positive	Negative	AG/GG		27	96	1.21 (0.67-2.18)	0.549
Negative	Negative	AA		13	23	2.43 (1.10-5.37)	0.042
Negative	Negative	AG/GG		9	34	1.14 (0.49-2.63)	0.827
*GSTT1*	*GSTM1*	*GSTP1* C341T		Case (193)	Control (514)	OR (95%IC)	P value
Positve	Positive	CC		57	198	1	
Positive	Positive	CT/TT		8	22	1.26 (0.53-2.98)	0.645
Negative	Positive	CC		28	49	1.98 (1.14-3.44)	0.017
Negative	Positive	CT/TT		2	6	1.15 (0.22-5.89)	1
Positive	Negative	CC		66	163	1.40 (0.93-2.12)	0.116
Positive	Negative	CT/TT		8	19	1.46 (0.60-3.51)	0.470
Negative	Negative	CC		22	50	1.52 (0.85-2.73)	0.162
Negative	Negative	CT/TT		2	7	0.99 (0.20-4.91)	1
*GSTT1*	*GSTM1*	*GSTP1* A313G*	*GSTP1* C341T	Case (178)	Control (514)	OR (95%IC)	P value
Positve	Positive	AA	CC	25	94	1	
Positive	Positive	AA	CT/TT	4	2	7.52 (1.30-43.45)	0.025
Positive	Positive	AG/GG	CC	24	104	0.86 (0.46-1.62)	0.749
Positive	Positive	AG/GG	CT/TT	4	20	0.75 (0.23-2.40)	0.784
Negative	Positive	AA	CC	16	21	2.86 (1.30-6.28)	0.01
Negative	Positive	AA	CT/TT	1	1	3.76 (0.22-62.28)	0.385
Negative	Positive	AG/GG	CC	12	28	1.61 (0.71-3.61)	0.281
Negative	Positive	AG/GG	CT/TT	1	5	0.75 (0.08-6.73)	1
Positive	Negative	AA	CC	38	85	1.68 (0.93-3.01)	0.106
Positive	Negative	AA	CT/TT	4	1	15.04 (1.60-140.68)	0.01
Positive	Negative	AG/GG	CC	23	78	1.10 (0.58-2.10)	0.870
Positive	Negative	AG/GG	CT/TT	4	18	0.83 (0.25-2.69)	1
Negative	Negative	AA	CC	12	23	1.96 (0.85-4.48)	0.118
Negative	Negative	AA	CT/TT	1	0	11.11 (0.43-281.37)	0.216
Negative	Negative	AG/GG	CC	8	27	1.11 (0.45-2.75)	0.817
Negative	Negative	AG/GG	CT/TT	1	7	0.53 (0.06-4.57)	1

Odds Ratio (OR) adjusted for age, gender, smoking habit and alcohol consumption

**Table 3:** Haplotype Frequency Distribution of *GSTP1 *Polymorphisms Gene between Case and Control Groups

A313G / C341T *GSTP1* polymorphisms			
Haplotype	HNSCC (%)	Control (%)	*P*- value
A-C	72.0	65.0	0.013
G-C	23.0	30.0	0.015
G-T	3.0	5.0	0.085
A-T	3.0	0.9	0.019

**Table 4:** *GSTT1, GSTM1*, A313G *GSTP1* and C341T *GSTP1* in Relation to the Primary Sites of Head and Neck Cancer Tumors

	Oral cavity			Larynx			Pharynx		
	n = 87 (44.0%)			n = 64 (32.0%)			n = 37 (19.0%)		
	n (%)	OR (95% CI)	p	n(%)	OR (95% CI)	p	n (%)	OR (95% CI)	p
GSTM1									
Positive	43 (49.0)	Reference		33 (51.0)	Reference		19 (51.0)	Reference	
Negative	44 (51.0)	0.98 (0.53-1.83)	0.955	31 (49.0)	1.02 (0.51-2.04)	0.945	18 (49.0)	0.78 (0.36-1.73)	0.545
GSTT1									
Positive	63 (72.0)	Reference		48 (75.0)	Reference		27 (73.0)	Reference	
Negative	24 (28.0)	0.83 (0.41-1.67)	0.596	16 (25.0)	0.77 (0.35-1.70)	0.513	10 (27.0)	0.97 (0.40-2.38)	0.947
A313G *GSTP1*^a^									
AA	46 (57.5)	Reference		28 (50.0)	Reference		22 (59.0)	Reference	
AG/GG	34 (42.5)	0.89 (0.48-1.67)	0.727	28 (50.0)	1.47 (0.74-2.93)	0.269	11 (41.0)	0.66 (0.29-1.48)	0.312
C341T *GSTP1*^b^									
CC	76 (90.5)	Reference		57 (90.5)	Reference		33 (91.6)	Reference	
CT	09 (9.5)	1.07 (0.39-2.92)	0.899	06 (9.5)	0.48 (0.14-1.65)	0.243	03 (8.4)	1.44 (0.43-4.82)	0.554

Odds Ratio (OR) adjusted for age, gender, smoking habit and alcohol consumption. ^a^Amplification was possible for 80, 56 and 33 patients for oral cavity, larynx and pharynx, respectively.^ b^Amplification was possible for 85, 63 and 36 patients for oral cavity, larynx and pharynx, respectively.

**Table 5:** Clinical-Histopathological Features in Relation to *GSTT1, GSTM1*, A313G *GSTP1* and C341T *GSTP1 *Polymorphisms in Patients with Head and Neck Cancer

	Tumor extension (191)				Regional lymph node involvement (195)							
	T1/T2 n (%)	T3/T4 n (%)	OR + (95% CI)	p	N=0 n(%)	N≥1 n(%)	OR+ (95% CI)	p	Early n (%)	Advanced n (%)	OR+ (95% CI)	p
*GSTM1*												
Positive	40 (51.3)	56 (49.5)	1		48 (44.8)	48 (54.5)	1		31 (50.0)	64 (48.5)	1	
Negative	38 (48.7)	57 (50.5)	1.06 (0.57-1.97)	0.860	59 (55.2)	40 (45.5)	0.74 (0.40-1.38)	0.350	31 (50.0)	68 (51.5)	1.15 (0.59-2.24)	0.673
*GSTT1*												
Positive	55 (70.5)	82 (72.6)	1		78 (72.8)	61 (69.3)	1		44 (70.9)	95 (71.9)	1	
Negative	23 (29.5)	31 (27.4)	0.82 (0.41-1.65)	0.580	29 (27.2)	27 (30.7)	1.23 (0.62-2.48)	0.550	18 (29.1)	37 (28.1)	1.22 (0.57-2.63)	0.605
A313G *GSTP1*^a^												
AA	42 (59.0)	55 (54.4)	1		55 (57.9)	46 (56.0)	1		34 (60.7)	67 (55.8)	1	
AG/GG	29 (41.0)	46 (45.6)	1.16 (0.62-2.16)	0.650	40 (42.1)	36 (44.0)	1.13 (0.61-2.11)	0.700	22 (39.3)	53 (44.2)	1.05 (0.53-2.06)	0.896
C341T *GSTP1*^b^												
CC	71 (92.0)	98 (89.0)	1		91 (80.5)	80 (91.0)	1		56 (91.8)	114 (88.4)	1	
CT	06 (8.0)	12 (11.0)	0.93 (0.34-2.54)	0.890	12 (19.5)	08 (9.0)	0.72 (0.26-2.00)	0.530	05 (8.2)	15 (11.6)	1.27 (0.40-3.98)	0.685

Odds Ratio (OR) adjusted for age, gender, smoking habit and alcohol consumption. ^a^Amplification was possible for 172, 177 and 176 patients for tumor extension, regional lymph node involvement and TNM, respectively.^ b^Amplification was possible for 187, 191 and 190 patients for tumor extension, regional lymph node involvement and TNM, respectively

**Table 6:** *GSTT1, GSTM1*, A313G *GSTP1* and C341T *GSTP1* Polymorphisms in Relation to Recurrence and Survival of Patients with Head and Neck Cancer

Polymorphisms	Relapse (12 months)	*P-* value Log Rank	Survival (12 months)	*P-* value Log Rank
*GSTT1*				
Positive	23.0%	0.467	85.0%	0.135
Negative	30.0%		72.0%	
*GSTM1*				
Positive	26.0%	0.717	85.0%	0.314
Negative	22.0%		80.0%	
A313G *GSTP1*				
AA	24.0%	0.450	78.0%	0.487
AG/GG	23.0%		88.0%	
C341T *GSTP1*				
CC	25.0%	0.164	83.0%	0.566
CT/TT	12.0%		82.0%	

## Discussion

The present study confirmed data from the literature, that report the predominance of cases between the fourth and sixth decade of life, and that smoking and alcohol consumption is strongly related to increased risk of developing this type of cancer (Choudhury et al., 2015; Rodrigues-Fleming et al., 2018). The studies carried out in Northeastern Brazil evidenced increased risk of oral cancer in patients presenting smoking and alcohol consumption synergism compared to non-synergetic consumption of cigarettes and alcohol (Andrade et al., 2015; Dias et al., 2017). 

Regarding, the *GST* gene family polymorphisms, the *GSTT1* and *GSTM1 *null genotype was not associated with the HNSCC carcinogenesis. Several studies have evaluated the association between these polymorphisms and head and neck cancer, however, the results are inconsistent (Kweon et al., 2014; He et al., 2016; Singh and Ghosh, 2019). A recent meta-analysis evaluated the effects of *GSTT1, GSTM1* null genotypes on the risk of oral cancer in the Chinese population, this study included 1,306 oral cancer cases and 1,484 controls and did not find a significant association for the two evaluated polymorphisms. In another study, the stratified analysis by ethnicity demonstrated significant risks for *GSTM1 *null genotypes and head and neck cancer among Asians (OR = 1.39, 95% CI = 1.27-1.53; P = 0.000), but not in Caucasians (OR = 0.99, 95% CI = 0.83-1.18; P = 0.677), thus corroborating our results (Li et al., 2018). 

The GSTT1 null genotype was less frequent among the investigated patients and healthy individuals and may indicate a reduced risk for cancer development. Similarly, studies on patients with HNSCC, such as the study carried in Brazilian population observed a lower frequency of the *GSTT1* null genotype (16.8%) in healthy subjects (Brunialti, 2009), while Silva et al. found a higher frequency of the *GSTT1* positive genotype among healthy individuals (24.1%) (Silva et al., 2014). 

*GSTT1 d*eletion leads to decreased ability to detoxify carcinogens efficiently and thus may increase ESCC risk. It has been consistently observed that those with homozygous *GSTT1* null genotypes are at a higher risk of cancer, which supports the involvement of *GSTT1 *polymorphisms in ESCC carcinogenesis (Makhdoomi et al., 2015). As the effect of any single gene probably has a limited impact on cancer risk, the combination of certain genotypes may be more effective. In this study, no association was found for this genotype alone, but we were able to demonstrate that the* GSTT1*null + *GSTM1* positive association increased the risk for HNSCC. A recent study from our research group demonstrated a protective association of GSTT1 null genotype and colorectal cancer (OR=0.65, 95% CI=0.43-0.98, P=0.037), which may justify our meeting for this genotype alone (Rodrigues-Fleming et al., 2018). 

Considering *GSTP1* polymorphisms, no association was possible between the *GSTP1*-C341T and the risk for HNSCC. However, this association was confirmed for the *GSTP1*-313 A/G + G/G genotypes polymorphism, where at least one polymorphic allele present confers reduced risk for head and neck cancer. The study carried out by Russo et al. (Russo et al., 2013), also in a Brazilian population with HNSCC, indicated that A313G *GSTP1 *AG/GG genotypes were associated with reduced risk for developing HNSCC, agreeing with the associations observed in the present study for this polymorphism. 

Genotypic frequencies for the A313G and C341T *GSTP1* polymorphisms were in Hardy-Weinberg equilibrium in both groups (p > 0.05). The A313G/C341T *GSTP1 *haplotypes (Table 3) revealed a higher frequency of the two wild alleles (A-C) in the case group than in the control group (72.0 and 65.0, respectively; p = 0.013). Similarly, a higher frequency of the A-T haplotype was also found in the case group than in the control group (3.0 and 0.9, respectively; p = 0.019). However, the opposite was observed for the G-C haplotype, which was more frequent in the control group than in the case group (23.0 and 30.0, respectively; p = 0.015).

The present study failed to statistically establish these associations and indicates that studies with larger sample numbers may better demonstrate the relation between the response to the chemotherapy and GSTs polymorphisms (Ruwali et al., 2009). On the other hand, the Indian population has demonstrated an association between null *GSTM1 *genotypes and toxicity observed due to cisplatin (Dhawan et al., 2013).

Our findings did not indicate a relationship between overall survival and the studied genotypes. The Brazilian study corroborates the findings related to survival and the *GSTP1* gene, but that study investigated the expression of this gene (Soares et al., 2017). Besides, reports of a trend towards significance (p=0.053) between the *GSTT1 *deletion polymorphism and progression-free survival in gastroesophageal adenocarcinoma are also found in the literature (Goekkurt et al., 2009).

The association of radiotherapy and/or chemotherapy in HNSCC treatment is evidenced by the literature and is indicated when it is impossible to perform surgery at the cancer site, as in the case of advanced tumors that affect important regions. Also, advanced tumors are more frequent when observing outpatient routine (Ruwali et al., 2009), as well as the findings obtained in the present study. Studies report that late HNSCC diagnosis can occur due to the absence of symptoms in most initial lesions, as well as due to lack of knowledge concerning differential diagnoses by some health professionals, contributing to late diagnosis with advanced-stage tumors (T3 and T4), already requiring radiotherapy and/or chemotherapy (Machiels et al., 2014).

In the present study, it was not possible to establish associations between primary anatomical site, clinical-pathological tumor features, relapse-free time and survival of HNSCC patients. To the primary anatomic site, a previous study in Brazilian HNSCC patients found no association of the *GSTT1 *null genotype with increased risk for larynx and decreased risk for pharynx cancers, thus, indicating that this polymorphism may modulate the risk to develop HNSCC at this anatomical site. The same study also found no association between the investigated *GST* polymorphisms with other clinical-pathological tumor features (Russo et al., 2013).

The predisposition to head and neck cancer is multifactorial and results from the interaction between allelic variant genes and environmental factors, such as old age, eating habits, and smoking and drinking habits. Therefore, the findings regarding the modulation of cancer susceptibility in the presence of the analyzed polymorphisms reinforce the influence on the etiology of the disease, although they do not influence patient survival. These results may contribute to the understanding of the mechanisms involved in the carcinogenesis of head and neck cancer. In conclusion, the present study indicates that age ≥59, male sex, smoking and drinking habits are risk factors for HNSCC development. The presence of the *GSTM1* null genotype is associated with increased risk for HNSCC, while the *GSTT1* null genotype and the *A313G GSTP1* polymorphism contribute to decreased risk for this tumor type. The *GSTM1*, *GSTT1* and *GSTP1* gene polymorphisms are not associated with tumor site, tumor extension, regional lymph node involvement, and tumor progression. Polymorphisms are also not associated with relapse-free time or survival time of patients treated with chemotherapy and/or radiotherapy.

*Ethics statement*

All the procedures performed in this study involving human participants were in accordance with the norms in force of the Ethics Committee on Human Research of our institution in Brazil CEP (Committee of Ethics and Research) / CONEP (National Commission of Ethics in Research) and São José do Rio Preto School of Medicine, (FAMERP), No. 697.896 and patients gave their informed consent before the material was obtained for use in the study. This article does not contain animal studies by any of the authors.
